# HM-Chromanone Alleviates Hyperglycemia by Activating AMPK and PI3K/AKT Pathways in Mice Fed a High-Fat Diet

**DOI:** 10.3390/nu16223972

**Published:** 2024-11-20

**Authors:** Jae-eun Park, Jeong Yoo, Ji-sook Han

**Affiliations:** 1 Department of Hotel Baking Technology, Busan Health University, Busan 49318, Republic of Korea; jepark@bhu.ac.kr; 2 Department of Food Science and Nutrition, Kimchi Research Institute, Pusan National University, Busan 46241, Republic of Korea; jessica19991@naver.com

**Keywords:** hyperglycemia, diabetes mellitus, insulin resistance, HM-chromanone, AMPK

## Abstract

Objectives: We investigated potential antihyperglycemic effects of HM-chromanone (HMC), a homoisoflavonoid isolated from *Portulaca oleracea*, in mice fed a high-fat diet (HFD). Methods: Five-week-old male C57BL/6J mice (*n* = 24) were divided into three groups: controls, mice fed an HFD (11 weeks), and HFD-fed mice receiving HMC supplementation (8 weeks). Various analyses assessed liver and skeletal muscle proteins, pancreatic β-cell histology, blood glucose and HbA1c levels, and homeostatic index of insulin resistance (HOMA-IR). Results: HMC supplementation significantly reduced fasting blood glucose and postprandial blood glucose levels in HFD-fed mice. HbA1c and serum insulin levels reduced significantly, and HOMA-IR improved. Compensatory β-cell hyperplasia was reduced, and pancreatic β-cell function improved. AMP-activated protein kinase (AMPK) was significantly activated in skeletal muscle and liver tissues. IRS-1tyr612 expression increased significantly. PI3K activation and Akt phosphorylation in skeletal muscles improved insulin signaling. Forkhead box protein O1 phosphorylation increased through hepatic AMPK activation. Phosphoenolpyruvate carboxykinase and glucose-6-phosphatase expression was inhibited. Glycogen synthase kinase 3β phosphorylation increased. Conclusions: HMC supplementation alleviated hyperglycemia by activating the AMPK and PI3K/Akt pathways in skeletal muscles and the AMPK pathway in the liver of HFD-fed mice.

## 1. Introduction

A high-fat diet affects glucose and lipid metabolism, which causes obesity and insulin resistance and alters pancreatic β-cell function [1]. Collectively, this results in hyperglycemia, a hallmark of type 2 diabetes mellitus [2,3]. Type 2 diabetes is a modern health problem with a rapidly increasing prevalence that has attracted worldwide attention owing to its associated morbidity [4]. Thus, it is important for people with diabetes to reduce hyperglycemia via treatment that can improve insulin resistance and pancreatic β-cell function [5].

In the course of a high-fat diet, lipids accumulate in the liver and in adipose tissue, ultimately leading to obesity [6]. During this process, the secretion of free fatty acids increases. Fatty acids interfere with the function of insulin receptors and signaling proteins in insulin-sensitive tissues, which lowers the sensitivity of insulin signaling and consequently causes insulin resistance [7]. In the early stage of diabetes, insulin resistance is associated with impaired glucose-uptake into cells and with pancreatic β-cell compensatory responses, including increased insulin biosynthesis and secretion [8,9]. As insulin resistance persists, more insulin is secreted to maintain normal blood glucose levels, causing hyperplasia and the dysfunction of pancreatic β cells [10,11].

Insulin resistance is caused by a defect in insulin signaling that inhibits glucose uptake into target cells [12]. Pathways involved in glucose uptake by target cells include the phosphatidylinositol 3-kinase/protein kinase B (PI3K/Akt) and AMP-activated protein kinase (AMPK) pathways. AMPK is a major regulator of cellular metabolism and is involved in glucose metabolism [13]. In skeletal muscles, the activation of AMPK results in the translocation of intracellular glucose transporter type 4 (GLUT4) from the cytosol to the plasma membrane to stimulate glucose uptake, resulting in the alleviation of hyperglycemia [14]. In the PI3K/Akt pathway, insulin activates tyrosine kinase when it binds to the insulin receptors present on the membranes of target cells [15]. The activated tyrosine kinase phosphorylates the tyrosine residue of the insulin receptor substrate (IRS), which in turn activates PI3K and phosphorylates Akt. The phosphorylated Akt also translocates GLUT4 to the plasma membrane to stimulate glucose uptake into the target cells [16]. However, if a serine residue is attached to the IRS, normal signal transduction does not occur, resulting in insulin resistance [17].

AMPK activation inhibits glucose production and is involved in glycogen synthesis [18,19]. In hepatic metabolism, AMPK activation results in the phosphorylation of forkhead box protein O1 (FOXO1), a transcription factor that regulates gluconeogenesis [20]. pFOXO1 reduces the expression of phosphoenolpyruvate carboxykinase (PEPCK) and glucose-6-phosphatase (G6Pase), thereby suppressing glucose production in the liver [21,22]. Activated AMPK also phosphorylates glycogen synthase kinase 3β (GSK3β), which is then responsible for maintaining glucose homeostasis through its activation of glycogen synthase (GS), a target of glycogen synthesis [23]. Thus, hyperglycemia can be ameliorated by reducing gluconeogenesis and increasing glycogen synthesis through AMPK-mediated PEPCK/G6Pase and GSK3β regulation.

Existing anti-diabetic drugs are prone to adverse effects, creating a need for the development of natural antidiabetic agents that reduce these side effects. One potential source is *Portulaca oleracea* L., a plant that has been used as a traditional food and medicine in Asian since ancient times [24,25]. Many studies have shown that *P. oleracea* exhibits the therapeutic effects, including anti-oxidant, neuroprotective, and anti-diabetes effects [24,26,27]. HMC can be isolated from *P. oleracea* and belongs to the homoisoflavonoid subclass of flavonoids, possessing a sappanin-like structure with a 3-benzylchroman backbone [28]. Previous studies have reported that HMC produces anti-obesity and anti-diabetic effects [29,30,31]. However, an anti-hyperglycemic effect of HMC has not been demonstrated in mice fed a high-fat diet. Therefore, the aim of this study was to investigate whether HMC alleviates hyperglycemia in mice fed a high-fat diet and, if so, to assess which pathways are involved.

## 2. Materials and Methods

### 2.1. Preparation of Material

**Figure 1:**
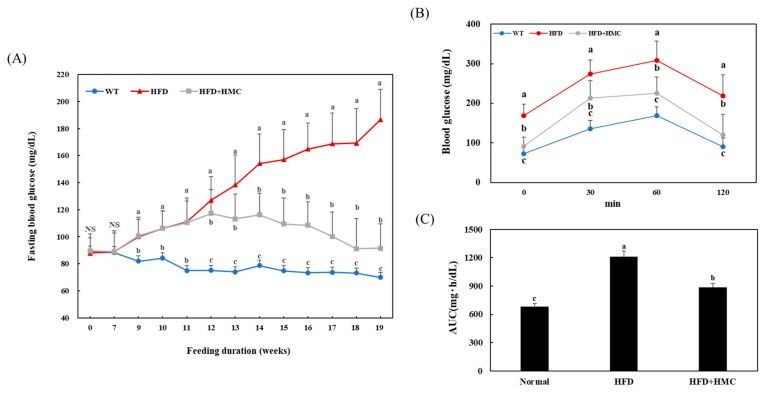
HMC improves high blood glucose in mice fed a high-fat diet. Normal group: C57BL/6J wild-type mice (*n* = 8) fed a normal chow diet; HFD group: C57BL/6J wild-type mice (*n* = 8) fed a high-fat diet; HFD + HMC group: C57BL/6J wild-type mice (*n* = 8) fed a high-fat diet and HM-chromanone 50 mg/kg body weight. (**A**) Fasting blood glucose level. (**B**) Oral glucose tolerance test level. (**C**) Area under the curve level. Each value is expressed as the mean ± SDE (*n* = 8). NS: not significant. ^a–c^ Values with different superscript letters are significantly different (a: *p* < 0.05, b: *p* < 0.01, c: *p* < 0.001) based on Bonferroni’s contrast test when using two-way ANOVA.

*Portulaca oleracea* plants were purchased from Hongcheon Hyosung Food Inc. (Gangwon, Hongcheon, Republic of Korea). The extraction and isolation of HMC were performed following a protocol. Figure 1 shows the chemical structure of HMC.

### 2.2. Study Animals

C57BL/6J wild-type mice (5-week male, *n* = 24) were obtained from the Animal Company at the Central Research Institute (Seoul, Republic of Korea), acclimatized for one week, and then used for experiments. During acclimation, mice were provided a stable environment with 55% humidity and temperature of 24 °C. After a 1-week adaptation period, a control group (*n* = 8) was fed a normal chow diet while a high-fat diet (HFD) group (*n* = 16) was fed a high-fat diet for 11 weeks (60 kcal%; all feed was purchased from the Animal Company, Central Research Institute) to induce diabetes (Table 1). Two mice per cage were housed. Different treatments were implemented from week 11 to week 19. The HFD group was administered 0.9% NaCl solution orally, and the HMC group received an oral supplement of HMC at 50 mg/kg of body weight, once daily. At the termination of the experimental period, the mice were fasted for 12 h before being sacrificed under ether anesthesia. All animal handling and care procedures were conducted in accordance with current international laws and policies as per the Pusan National University (Busan, Republic of Korea) Guide for the Care and Use of Laboratory Animals (Approval no. PNU-2022-0112).

### 2.3. Tissue Collection

Skeletal muscle of the hindlimb [tibialis anterior (TA), gastrocnemius, and extensor digitorum longus] and liver tissues were collected. Frozen tissues were ground before metabolite extraction.

### 2.4. Western Blot Analysis

Western blot was performed on the protein extracts of the liver and skeletal muscle. Skeletal muscle and liver homogenates in buffer were centrifuged at 20,000× *g* (4 °C) for 15 min. Isolated proteins were blocked with 5% skim milk and 0.1% Tween20 in Tris-buffer for 60 min at room temperature. Blocked membranes were incubated with primary antibodies against AMPK, IRS-1ser307, IRS-1tyr612, PI3K, Akt, GLUT4, G6pase, FOXO1, G6Pase, PEPCK, GSK3β, and GS for 60 min (Abcam, Cambridge, UK). After washing the membrane, the secondary antibody was incubated for 60 min, and each antigen–antibody complex was visualized using a Western blotting detection reagent. Chemiluminescence was detected using an LAS-1000 Plus Analyzer (Fujifilm, Tokyo, Japan), and band density was measured using Image Analyzer (Multi Gauge V3.1, Fujifilm, Tokyo, Japan).

### 2.5. Blood Glucose Levels and Glycosylated Hemoglobin Levels

Normal group mice, HFD-group mice, and HMC-supplemented mice were fasted overnight. Fasted animals had no food intake for 12 h. After the overnight fast, mice were orally supplemented with soluble starch [2 g/kg body weight (bw)]. Blood samples were taken from the tail vein at 0, 30, 60 and 120 min. Collected blood glucose was measured using a glucometer (Roche Diagnostics GmbH, Mannheim, Germany). The areas under the curve (AUC) were calculated using the trapezoidal rule. A hemolyzed sample of anti-coagulated whole blood was used to measure glycated hemoglobin by the immunoturbidity method.

### 2.6. Serum Analysis

Serum insulin levels were measured using the mouse insulin ELISA Kit (Shibayagi, Gunma, Japan).

### 2.7. Pancreatic Histology and Morphometry

For the analysis of islet cytoarchitecture (i.e., the typical arrangement of beta- and non-beta-cells within the islet), 42 the pancreas sections were processed for dual immune fluorescence for insulin (using the anti-insulin, Dako, Carpinteria, CA, USA) and glucagon (using the anti-glucagon, Dako, USA) using a standard protocol and observed by confocal laser microscopy (LSM 510-Zeiss, Land Baden-Württemberg, Germany). For the evaluation of adipocyte infiltration within the pancreas, we used the same histological sections processed previously for HE staining and insulin immune peroxidase.

### 2.8. Statistical Analyzes

Data obtained from this study are expressed as the mean ± SEM using SPSS version 26.0 (IBM Corp., Armonk, NY, USA). Data obtained over time were analyzed using a repeated measures two-way ANOVA test. Post hoc comparisons between selected means were performed with Bonferroni’s contrast test when initial two-way ANOVA indicated statistical differences between experimental groups. The level of significance was set at ^a^: *p* < 0.05, ^b^: *p* < 0.01, ^c^: *p* < 0.001.

## 3. Results

### 3.1. Body Weight and Food Intake

Table 2 shows the weight gain, food intake, and water intake of mice during the experimental period. There was no significant difference in initial body weight between the HFD group (22.33 ± 1.20 g) and the HMC-supplemented group (22.73 ± 0.73 g). Final body weight was significantly lower in the HMC-supplemented group (42.24 ± 3.24 g) than in the HFD group (46.76 ± 3.10 g) (*p* < 0.05). Correspondingly, weight gain in the HMC-supplemented group (19.51 ± 4.18 g) was significantly lower than that in the HFD group (24.43 ± 3.51 g). The average food intake and water intake of the HFD group (2.94 ± 0.30 g and 2.82 ± 0.34 mL, respectively) were higher than those of the HMC-supplemented group (2.86 ± 0.34 g and 2.75 ± 0.21 mL, respectively).

### 3.2. Blood Glucose Levels

Figure 1A shows the effect of HMC on fasting blood glucose levels in HFD-fed mice. In the HMC-supplemented group, the mice were fed an HFD for up to 11 weeks but also received HMC as a supplement from week 11 to week 19. There was no significant difference in blood glucose levels between these two groups up to the 11-week mark. After 12 weeks, the HFD group continued to show an increase in blood glucose levels, as previously observed, but blood glucose levels in the HMC-supplemented group first stabilized and then gradually decreased. At the termination of the experiment (19 weeks), the fasting blood glucose level was 91.45 mg/dL in the HMC-supplemented group, which was significantly lower than the 186.75 mg/dL recorded for the HFD group. The postprandial blood glucose level of the HFD group increased to 274.00 mg/dL at 30 min, 308.56 mg/dL at 60 min, and then decreased to 218.44 mg/dL at 120 min (Figure 1B). This increase in postprandial blood glucose level was significantly ameliorated in the group supplemented with HMC, with readings of 213.55 mg/dL, 225.13 mg/dL, and 120.23 mg/dL at 30 min, 60 min, and 120 min, respectively. The AUC for the blood glucose response was 885.09 ± 141.89 mg·h/dL in the HMC-supplemented group, which was significantly lower than the 1211.95 ± 132.81 mg·h/dL in the HFD group (Figure 1C).

### 3.3. HbA1c, Serum Insulin, and HOMA-IR Levels

**Figure 2:**
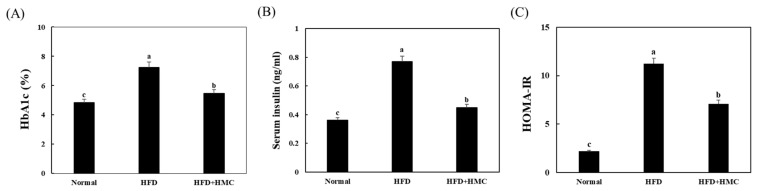
HM-chromanone improves HbA1c and insulin resistance in mice fed a high-fat diet. Normal group: C57BL/6J wild-type mice (*n* = 8) fed a normal chow diet; HFD group: C57BL/6J wild-type mice (*n* = 8) fed a high-fat diet; HFD + HMC group: C57BL/6J wild-type mice (*n* = 8) fed a high-fat diet and HM-chromanone 50 mg/kg body weight. (**A**) HbA1c level, (**B**) serum insulin level, and (**C**) homeostatic index of insulin resistance level. Each value is expressed as the mean ± SDE (*n* = 8). ^a–c^ Values with different superscript letters are significantly different (a: *p* < 0.05, b: *p* < 0.01, c: *p* < 0.001) based on Bonferroni’s contrast test when using two-way ANOVA.

The effect of HMC on HbA1c, serum insulin, and HOMA-IR levels in HFD-fed mice is depicted in Figure 2. The HbA1c level of the HFD group (7.25 ± 1.81%) was significantly higher than that of the control group (4.83 ± 0.52%). In the group receiving HMC supplementation, an HbA1c level of 5.46 ± 0.67% was significantly lower than that of the HFD group.

Serum insulin levels were 0.36 ± 0.02 ng/mL, 0.77 ± 0.09 ng/mL, and 0.45 ± 0.03 ng/mL in the control, HFD, and HMC-supplemented groups, respectively. The level in the HFD group was significantly higher than that in the control group; however, the mean serum insulin level in the HMC-supplemented group was significantly lower than that in the HFD group.

The HOMA-IR values were 2.2 ± 0.12 and 20.4 ± 2.56 in the control and HFD groups, respectively. However, the HOMA-IR value of the HMC-supplemented group, 7.1 ± 1.45, was significantly lower than that of the HFD group.

### 3.4. Pancreatic β-Cell Histology

**Figure 3:**
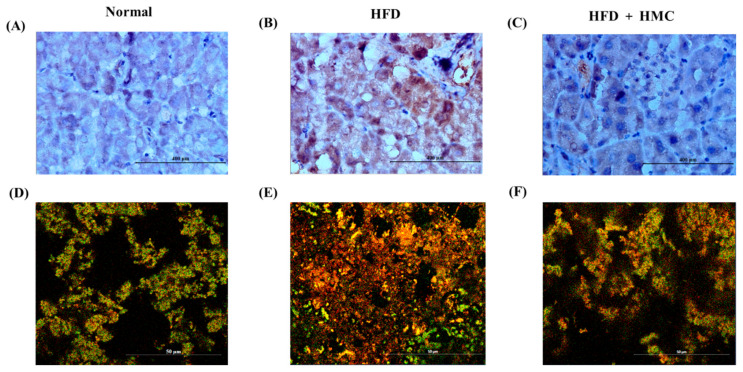
HMC improves the function of pancreatic β cell in mice fed a high-fat diet. Normal group: C57BL/6J wild-type mice (*n* = 8) fed a normal chow diet; HFD group: C57BL/6J wild-type mice (*n* = 8) fed a high-fat diet, HFD + HMC group: C57BL/6J wild-type mice (*n* = 8) fed a high-fat diet and HM-chromanone 50 mg/kg body weight. Micrographs of pancreatic tissue treated for insulin immunohistochemical staining (brown) (**A**–**C**) or double immunofluorescence for detection of insulin (red) and glucagon (green) (**D**–**F**). Images were obtained with light (**A**–**C**) or confocal laser microscopy (**D**–**F**). Scale bar: 400 μm (**A**–**C**), 50 μm (**D**–**F**).

The effect of HMC on the function of pancreatic β cells in mice fed on an HFD is shown in Figure 3. The immunohistochemical analysis recorded more insulin in the HFD group than in the control group. In contrast, the HMC-supplemented group showed significantly lower insulin levels than the HFD group. In the double immunofluorescence assay, more insulin was once again observed in the HFD group than in the normal group, and the insulin levels were once again lower in the HMC-supplemented group than in the HFD group.

### 3.5. Expression of pAMPK and pACC in the Skeletal Muscle

**Figure 4:**
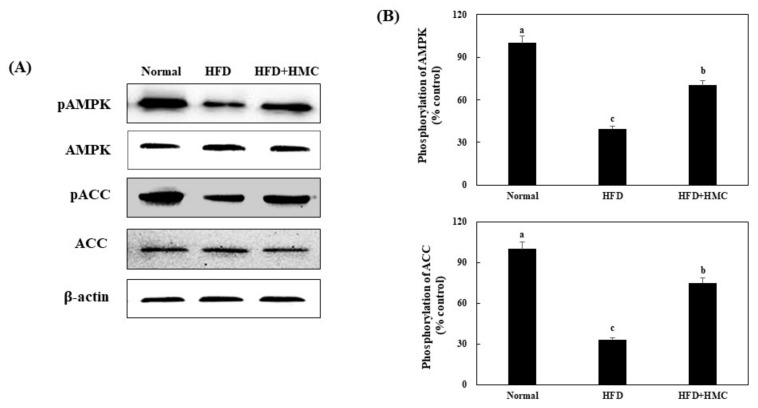
HMC activates AMPK in skeletal muscle of mice fed a high-fat diet. Normal group: C57BL/6J wild-type mice (*n* = 8) fed a normal chow diet; HFD group: C57BL/6J wild-type mice (*n* = 8) fed a high-fat diet; HFD + HMC group: C57BL/6J wild-type mice (*n* = 8) fed high-fat diet and HM-chromanone 50 mg/kg body weight. (**A**) pAMPK, AMPK, pACC, and ACC expression. (**B**) Expression levels of pAMPK and pACC. Each value is expressed as the mean ± SDE (*n* = 8). ^a–c^ Values with different superscript letters are significantly different (a: *p* < 0.05, b: *p* < 0.01, c: *p* < 0.001) based on Bonferroni’s contrast test when using two-way ANOVA.

The effects of HMC on pAMPK and pACC expression were investigated in the skeletal muscles of HFD-fed mice (Figure 4). Compared to the expression of pAMPK and pACC in the control group (100% for both), that in the HFD group was significantly decreased to 39.61% and 33.00%, respectively. On the other hand, HMC supplementation significantly increased the expression of pAMPK and pACC compared to that in the HFD group, reaching 70.23% and 75.00%, respectively. Collectively, these results indicated that HMC activated AMPK in the skeletal muscles of HFD-fed mice.

### 3.6. Expression of IRS-1, PI3K, AKT, and PM-GLUT4 in the Skeletal Muscle

**Figure 5:**
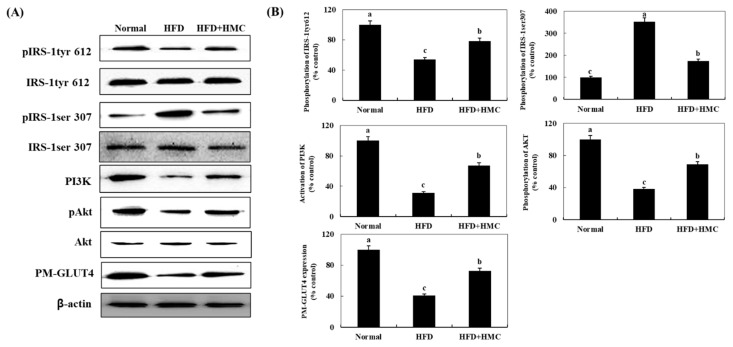
HMC improves insulin signaling in skeletal muscle tissue of mice fed a high-fat diet. Normal group: C57BL/6J wild-type mice (*n* = 8) fed a normal chow diet; HFD group: C57BL/6J wild-type mice (*n* = 8) fed a high-fat diet; HFD + HMC group: C57BL/6J wild-type mice (*n* = 8) fed a high-fat diet and HM-chromanone 50 mg/kg body weight. (**A**) pIRS-1tyr612, IRS-1tyr612, pIRS-1ser 307, PI3K, pAkt, Akt, and PM-GLUT4 expression. (**B**) Expression levels of pIRS-1tyr612, pIRS-1ser 307, PI3K, pAkt, and PM-GLUT4. Each value is expressed as the mean ± SDE (*n* = 8). ^a–c^ Values with different superscript letters are significantly different (a: *p* < 0.05, b: *p* < 0.01, c: *p* < 0.001) based on Bonferroni’s contrast test when using two-way ANOVA.

We investigated the effect of HMC on the expression of pIRS-1tyr612, pIRS-1ser307, PI3K, and pAKT, which are transcription factors involved in insulin signaling in the skeletal muscle of mice (Figure 5). The expression of pIRS-1tyr612 decreased from 100% in the control group to 53.69% in the HFD group. On the other hand, its expression in the HMC-supplemented group was significantly higher than that in the HFD group, at 78.25%. The expression of pIRS-1ser307 increased from 100% in the control group to 353.05% in the HFD group, compared with which a significantly decreased expression of 174.31% was recorded for the HMC-supplemented group. Similarly, the expression of PI3K, pAkt, and PM-GLUT4 decreased significantly from 100% for all in the control group to 31.27%, 38.12%, and 41.00%, respectively, in the HFD group; however, corresponding expression in the HMC-supplemented group showed significant increases of 67.35%, 69.00%, and 72.68%, respectively, compared to those in the HFD group.

### 3.7. Expression of Factors Related to the AMPK and Akt Pathways in the Liver

**Figure 6:**
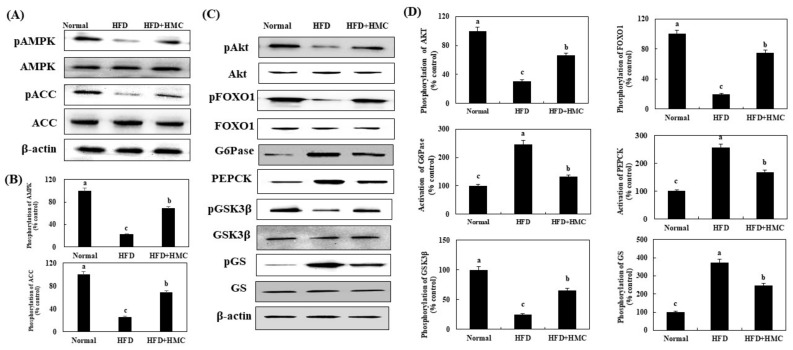
HMC improves AMPK and Akt/FOXO1 pathway in the liver of mice fed a high-fat diet. Normal group: C57BL/6J wild-type mice (*n* = 8) fed a normal chow diet; HFD group: C57BL/6J wild-type mice (*n* = 8) fed a high-fat diet; HFD + HMC group: C57BL/6J wild-type mice (*n* = 8) fed a high-fat diet and HM-chromanone 50 mg/kg body weight. (**A**) pAMPK, AMPK, pACC, and ACC expression. (**B**) Expression levels of pAMPK, AMPK, pACC, ACC. (**C**) pAkt, Akt, pFOXO1, FOXO1, G6Pase, PEPCK, pGSK3β, GSK3β, pGS, and GS expression. (**D**) Expression levels of pAkt, pFOXO1, G6Pase, PEPCK, pGSK3β, and pGS. Each value is expressed as the mean ± SDE (*n* = 8). ^a–c^ Values with different superscript letters are significantly different (a: *p* < 0.05, b: *p* < 0.01, c: *p* < 0.001) based on Bonferroni’s contrast test when using two-way ANOVA.

The effect of HMC on the expression of pAMPK, pACC, pAkt, pFOXO1, G6Pase, PEPCK, pGSK3β, and pGS in the liver of mice fed with an HFD were investigated (Figure 6). The expression of pAMPK and pACC was significantly reduced from 100% in the control group to 22.00% and 26.28%, respectively, in the HFD group. In contrast, the expression of pAMPK and pACC was significantly higher in the HMC-supplemented group, at 69.00% and 68.61%, respectively, compared with the HFD group. Similarly, the expression of pAkt and pFOXO1 was significantly lower in the HFD group, 30.79% and 19.59%, respectively, than in the control group (100%). An ameliorative pattern was again observed in the HMC-supplemented group, in which the expression of pAkt and pFOXO1 (65.93% and 74.52%, respectively) was significantly higher than that in the HFD group. In addition, the expression of G6Pase and PEPCK were significantly increased to 247.18% and 256.25%, respectively, in the HFD group when compared to their expression in the control group (100%). However, these heightened figures were significantly reduced in the HMC-supplemented group, with the expression of G6Pase and PEPCK being recorded at 131.90% and 167.40%, respectively. The expression of pGSK3β decreased significantly from 100% in the control group to 24.64% in the HFD group, compared with which it was significantly higher in the HMC-supplemented group, at 65.57%. Inversely, the expression of pGS increased significantly from 100% in the control group to 372.28% in the HFD group, compared with which it was ameliorated with HMC treatment and significantly decreased to 246.95% in the HMC-supplemented group.

## 4. Discussion

The previous study investigated the effects of HMC on hyperglycemia and dyslipidemia in C57BL/KsJ-db/db mice [31]. However, an anti-hyperglycemic effect of HMC has not been demonstrated in mice fed a long-term high-fat diet to induce diabetes. Obesity is a key underlying cause of type 2 diabetes mellitus. A long-term HFD can cause obesity, impaired glucose tolerance, and insulin resistance, which are key factors in the pathogenesis of diabetes and lead to hyperglycemia [32,33,34]. This study investigated whether HMC isolated from *P. oleracea* could alleviate hyperglycemia in mice fed on a long-term HFD, and if so, by what pathway. The long-term HFD-induced obesity mouse model exhibits characteristics such as obesity-related insulin resistance, hyperglycemia, and hyperinsulinemia [35,36].

Diabetes mellitus patients must pay attention to the management of blood glucose levels. When the pancreas detects rising blood glucose levels, it secretes insulin to absorb the glucose into cells [37]. It also reduces glucose production in the liver and increases glycogen synthesis [38]. However, if insulin resistance develops, these metabolic processes progress abnormally and result in a hyperglycemic state. Therefore, the fasting blood glucose level is one of the diagnostic indicators of diabetes mellitus and blood glucose control status, with good control of fasting blood glucose being critical in the management of diabetes. In our study, the fasting blood glucose level in HMC-supplemented mice was significantly lower than that in the HFD group. The result confirms that HMC supplementation can reduce fasting blood glucose levels in HFD-fed mice. HMC is a saponin-type homoisoflavonoid. Homoisoflavonoids isolated from *Cucumis bisexualis* have similarly been shown to reduce fasting blood glucose levels [39], as has brazilin, a major homoisoflavonoid component found in the heartwood of Caesalpinia sappan L. [40]. Several studies have shown that homoisoflavonoids are effective at controlling blood glucose levels [41], providing the basis for our hypothesis that HMC may similarly reduce fasting blood glucose levels in HFD-fed mice.

Postprandial blood glucose levels can more accurately reflect the blood glucose metabolic response and blood glucose control ability than fasting blood glucose levels [42]. Increases in blood glucose levels after a meal are also related to fasting blood glucose levels [43]. In the early stages of diabetes, fasting blood glucose levels are relatively well controlled, while postprandial blood glucose gradually increases [44]. If postprandial blood glucose levels consistently exceed a certain level, fasting blood glucose levels may also increase and diabetes symptoms may worsen. Therefore, improving postprandial hyperglycemia is an important aspect of diabetes treatment. Oral glucose tolerance tests were performed to measure the increase in postprandial blood glucose levels in our experimental mice. The HMC-supplemented group had significantly lower blood glucose levels than the HFD group at 30, 60, and 120 min, indicating that HMC supplementation may contribute to the reduction in postprandial hyperglycemia. HbA1c is an indicator of the long-term control of blood glucose via the chemical linking of hemoglobin and glucose molecules [45]. In patients with diabetes mellitus, the average lifespan of red blood cells is 120 days. The non-enzymatic and irreversible glycation of hemoglobin continues to occur in cells during this period, resulting in an increase in the concentration of HbA1c [46]. Many studies have reported that an increase in HbA1c concentration can worsen diabetes and cause retinopathy, neuropathy, and microvascular issues, among other complications [47]. In our study, mice that received HMC supplementation showed a significant decrease in HbA1c. This finding suggests that HMC supplementation may be effective for long-term blood glucose control.

When blood glucose levels increase, insulin is secreted from pancreatic β cells to promote glucose uptake and suppress glucose production in the muscles and liver [48]. However, when insulin resistance occurs due to the impairment of insulin signaling, compensatory hyperinsulinemia develops as a result of excessive insulin secretion in the body [49]. In this study, the insulin concentration in mice fed on an HFD was significantly increased as compensation for the lowered blood glucose levels, whereas the insulin concentration in mice receiving HMC supplementation was significantly decreased. These results suggest that the compensatory secretion of insulin may also be ameliorated as blood glucose levels are decreased via HMC. HOMA-IR is used to estimate insulin resistance and can be obtained using blood glucose and insulin concentrations.

When blood glucose rises, the pancreas attempts to control blood glucose by secreting more insulin, which may be successful initially, but over time, pancreatic β-cell function may deteriorate [50,51]. This continuous process progressively deteriorates the β-cell function, restricting glycemic control and ultimately leading to persistent hyperglycemia and progression to diabetes. To manage this disease, the protection of pancreatic β-cell function is therefore essential. In our immunohistochemical analysis, less insulin was detected in the pancreas of mice from the HMC-supplemented group than in that of mice from the HFD group. This finding was consistent with our blood insulin concentration results. We could therefore conclude that pancreatic β-cell function was protected in the HMC-supplemented group. A previous study has demonstrated that baicalein (5,6,7-Trihydroxyflavone) similarly protected pancreatic β-cell function and reduced blood glucose [52]. Genistein, an isoflavone, has also been shown to alleviate blood glucose and protect pancreatic β-cell function in diabetic mice [53]. Genistein contains hydroxyl groups at C5 and C7 of the A ring and a hydroxyl group at C4 of the C ring [54]. The mechanism of β-cell function protection in this compound was linked with the hydroxyl group at C5 of its A ring [55]. HMC similarly possesses hydroxyl groups at the C5 and C2 positions. We therefore propose that HMC may protect pancreatic β-cell function via its hydroxyl group at the C5 position.

Insulin signaling plays an important role in glycemic control [56]. Insulin binds to the insulin receptor, which induces phosphorylation of the IRS at tyrosine residues, activating downstream signaling molecules, such as PI3K and Akt, with activated Akt ultimately translocating GLUT4 to the plasma membrane [57]. This signaling stimulates glucose uptake into skeletal muscle cells and lowers blood glucose levels [58]. In our study, the expression of IRS-1tyr, PI3K, and Akt increased in the skeletal muscles of mice that received HMC supplementation; PM-GLUT4 expression was similarly increased in this group. This indicates the promotion of glucose uptake into skeletal muscle cells to reduce blood glucose levels. Additionally, the activation of the insulin-independent AMPK signaling pathway could also increase GLUT4 translocation, thereby promoting glucose uptake into cells and lowering blood glucose levels [59]. The phosphorylation of AMPK in skeletal muscle tissue was indeed significantly increased in the HMC-supplemented group, suggesting that HMC enhances glucose uptake by stimulating GLUT4 translocation to the plasma membrane through the PI3K and AMPK pathways in skeletal muscle. Biochanin A (5,7-dihydroxy-4′-methoxyisoflavone) was previously reported to improve the insulin signaling and stimulate glucose uptake [60]. This compound has hydroxyl groups bonded to C5 and C7 and a methoxy group bonded to C4′. Flavonoid compounds with multiple hydroxyl groups have a much stronger activity in stimulating glucose uptake [61]. Additionally, the C3 methine group was shown to affect AMPK activation in homoisoflavonoids [62]. As already noted, HMC contains hydroxyl groups at the C5 and C7′ positions, but also a methine group at the C3 position. These three functional groups in HMC seem to be involved in promoting glucose uptake by improving insulin signaling and activating AMPK, resulting in lowering blood glucose levels.

Furthermore, AMPK is a known target factor that regulates gluconeogenesis and glycogen synthesis in the liver [63]. AMPK activation induces FOXO1 phosphorylation, which regulates the expression of gluconeogenesis-related genes in the liver [64]. Phosphorylation of FOXO1 inhibits G6Pase and PEPCK, thereby reducing hepatic gluconeogenesis and contributing to blood glucose control [65]. Additionally, AMPK activation is involved in glycogen synthesis by reducing the phosphorylation of GS and inactivating GSK3β [66]. In this study, the phosphorylation of FOXO1 was increased due to AMPK activation, and the expression of PEPCK and G6pase was decreased in the HMC-supplemented group. Additionally, GSK3β was phosphorylated and GS phosphorylation was significantly inhibited. These results demonstrate that HMC reduces gluconeogenesis by activating AMPK and increasing glycogen synthesis in the liver. Previous studies have reported that hesperetin, a 3′,5,7-trihydroxy-4′ methoxy flavanone, inhibits hepatic gluconeogenesis and increases glycogen synthesis [67]. The chemical structure of hesperetin has one methoxy group and three hydroxy groups at C3′, C5, and C7. In addition, nobiletin (2-(3,4-dimethoxyphenyl)-5,6,7,8-tetramethoxy-4H-chromen-4-one) induces GSK3β phosphorylation through AMPK activation and promotes glycogen synthesis in the liver [68]. Nobiletin has two methoxy groups bonded to C3 and C4 on the phenyl ring and four methoxy groups located at C5, C6, C7, and C8 of the chromen structure. One study reported that the methoxy group of flavonoid compounds affects glycogen synthesis [69]. Additionally, kaempferol inhibited glucose production by reducing PEPCK and G6Pase expression via AMPK activation [70]. Kaempferol has hydroxy groups at C3, C5, and C7; a double bond between C2 and C3 of the C ring; and an oxo group at C4. Again, the presence of a hydroxy group in flavonoid compounds and an oxo group at the 4 position have been associated with the inhibition of gluconeogenesis [71]. HMC has two hydroxy groups at C5 and C2′, one methoxy group at C7, and an oxo group at the 4 position. The authors therefore hypothesize that the two hydroxy, one methoxy, and 4-oxo functional groups of HMC contribute mechanistically to the promotion of glycogen synthesis and inhibition of gluconeogenesis in the liver.

## 5. Conclusions

HMC supplements significantly improved hyperglycemia in mice fed on an HFD. It increased IRS-1tyr612 phosphorylation and improved insulin resistance by activating PI3K/AKT and AMPK. It also significantly reduced gluconeogenesis by downregulating PEPCK and G6Pase through the AMPK/FOXO1 pathway in the liver. Additionally, HMC supplementation reduced compensatory β-cell proliferation and improved pancreatic β-cell function. Collectively, these data suggest that HMC can be applied to alleviate hyperglycemia by activating the AMPK and PI3K/Akt pathways in skeletal muscle and the AMPK pathway in the liver.

## Figures and Tables

**Table 1:** The dietary composition.

Composition (g/kg)	Normal Diet	High-Fat Diet
Corn starch	315	0
Sucrose	350	89
Maltodextrin	35	162
Fiber	50	65
Casein	200	258
L-cystine	3	4
Soybean oil	25	32
Lard	20	316
Mineral mixture	10	13
Vitamin mixture	10	13
Potassium citrate	16.5	21
Calcium phosphate	5.5	7
Dicalcium phosphate	13	17
Choline bitartrate	2	2.5

**Table 2:** HMC decreases body weight and food intake in mice fed a high-fat diet.

	Normal	HFD	HFD + HMC
Body weight			
Initial weight (g)	22.18 ± 0.51 ^NS^	22.33 ± 1.20	22.73 ± 0.73
Final weight (g)	29.32 ± 2.25 ^c^	46.76 ± 3.10 ^a^	42.24 ± 3.24 ^b^
Weight gain (g)	7.14 ± 2.41 ^c^	24.43 ± 3.51 ^a^	19.51 ± 4.18 ^b^
Average food intake (AFI) (g/day)	4.07 ± 0.28 ^a^	2.94 ± 0.30 ^b^	2.86 ± 0.34 ^c^
Average water intake (AWI) (mL/day)	4.11 ± 1.05 ^a^	2.82 ± 0.34 ^b^	2.75 ± 0.21 ^b^

Normal group: C57BL/6J wild-type mice (*n* = 8) fed a normal chow diet; HFD group: C57BL/6J wild-type mice (*n* = 8) fed a high-fat diet; HFD + HMC group: C57BL/6J wild-type mice (*n* = 8) fed a high-fat diet and HM-chromanone 50 mg/kg body weight. Each value is expressed as the mean ± SDE (*n* = 8). NS: not significant. ^a–c^ Values with different superscript letters are significantly different (a: *p* < 0.05, b: *p* < 0.01, c: *p* < 0.001) based on Bonferroni’s contrast test when using two-way ANOVA.

## Data Availability

The original contributions presented in the study are included in the article, further inquiries can be directed to the corresponding author.
